# Lethal Lust: Suicidal Behavior and Chemsex—A Narrative Review of the Literature

**DOI:** 10.3390/brainsci13020174

**Published:** 2023-01-20

**Authors:** Martina Strasser, Theresa Halms, Tobias Rüther, Alkomiet Hasan, Marcus Gertzen

**Affiliations:** 1 Psychiatry and Psychotherapy, Faculty of Medicine, University of Augsburg, Geschwister-Schönert-Str. 1, 86156 Augsburg, Germany; 2 Department of Psychiatry, Ludwig-Maximilians-University Hospital Munich, 80336 Munich, Germany

**Keywords:** chemsex, suicide, sexualized drug use, slamsex, men who have sex with men, mental health

## Abstract

Chemsex is described as the use of certain drugs—commonly methamphetamine, gamma-butyrolactone (GBL)/gammahydroxybutyrate (GHB), and mephedrone—before or during planned sexual activity primarily among men who have sex with men (MSM). Evidence shows that MSM who engage in chemsex are at increased risk of physical harm, such as sexually transmittable infections (STIs), and are more likely to experience mental health symptoms. To further assess this, we reviewed the recent literature to evaluate whether the psychological impact of chemsex behavior includes suicidal ideation and suicidal attempts. Pubmed/MEDLINE was searched for articles reporting suicidal ideation and behavior among chemsex users with the terms “chemsex”, “sexualized drug use”, “suicide”, and “mental health”. Twelve articles (three case reports and nine cross-sectional studies) were included in the final narrative review. Overall, we retrieved mixed results regarding the relationship between chemsex practice and suicidality outcomes. Considering the inhomogeneous nature of the studies, the findings indicate that suicidality could be an issue of concern among MSM in general but among chemsex users in particular. Possible risk factors for suicidality among chemsex participants may include adversities experienced due to one’s sexual orientation and an increased risk for HIV and other STI infections and the resulting negative impact on mental well-being. These aspects warrant further investigations.

## 1. Introduction

The term “chemsex”, a subform of sexualized substance use, was first described in the United Kingdom as the intentional use of certain substances immediately before or during sexual activities primarily among men who have sex with other men (MSM) [1,2]. While most men who engage in chemsex administer drugs non-intravenously, a smaller proportion of chemsex participants use intravenous, a phenomenon known as “slamsex” or “slamming” [3]. Four substances are commonly associated with chemsex: Methamphetamine, mephedrone, and gammahydroxybutyrate (GHB)/gammabutyrolactone (GBL) [1]. These drugs are often used in combination to facilitate, prolong, or intensify sexual experiences [4,5], as well as to enhance confidence and perceptions of intimacy [6]. However, other authors have expanded the definition of chemsex by including other drugs [7]: Psychoactive drugs and sex enhancers such as ketamine, alkyl nitrites (poppers), or erectile dysfunction agents [7]. To our knowledge, no consensus definition of the term “chemsex” is available in the literature.

One approach to explaining why MSM have been found to be at high risk for this type of substance use is detailed by Meyer’s Minority Stress Model [8]. This model emphasizes that increased exposure to minority stress (i.e., prejudice and discrimination found within minority communities) contributes to the disparity between dominant and minority values [8]. Therein, according to this concept, the constant stressors experienced by MSM may lead to reduced mental health and perpetuate negative coping mechanisms including harmful substance use [8]. Previous studies have shown that lesbian, gay, and bisexual (LGB-) people may be at higher risk of mental disorders, harmful substance use and dependence, and deliberate self-harm when compared to heterosexual people [9]. Gay and bisexual men reported higher prevalence rates of anxiety, depression, and licit and illicit drug use than the general population [10]. A systematic review of 25 studies estimated a twofold increased risk of suicide attempts in the preceding year in LGB people, and a fourfold increased risk in gay and bisexual men over a lifetime [9]. Authors demonstrated that alcohol and other harmful substance use and dependence over 12 months was 1.5 times higher than in heterosexual people [9]. In turn, it is well known that people with mental health disorders, including substance use disorder, are at increased risk of suicide [11,12]. A recent review providing data on the harmful use of new psychoactive substances and the effects on mental health and suicidality issues confirms this assumption with regard to new synthetic drugs such as mephedrone [13].

Therefore, gay or bisexual men who engage in chemsex may be, according to Stuart [1], a small but significant and particularly vulnerable population of men. Recent reviews of the literature indicate that chemsex is associated with several health implications [5,14,15]. MSM practicing chemsex showed enhanced high-risk sexual activities such as engaging in lengthy, condomless “sex sessions” with a larger number of casual partners. This increases the prevalence of sexually transmittable infections (STIs) and the transmission of viral infections such as those caused by HIV and hepatitis C, among others [5,16]. For men who practice slamsex, the risk of infection increases clearly if the equipment is shared. Further, the risk of infections and other adverse health effects may be increased due to chemsex users’ reluctance to use support and information services [17]. With regard to mental health, the majority of available studies revealed associations between sexualized drug use and mental burden [15,18]. Íncera-Fernández et al. demonstrated in a recent review that MSM who combine drugs with sex report higher levels of depression, anxiety, and dependence symptoms, although some studies did not confirm these results [18]. Additionally, an online survey of 1648 MSM in the UK showed a significant association between sexualized substance use and low life satisfaction, as well as sexual self-efficacy [19]. Furthermore, a recently published systematic review revealed a significant link between the practice of chemsex and the development of psychosis [20]. Both the mediating role of drug use and the impact of several risk factors, such as loneliness and belonging to an ethnic minority, were identified. The relationship between chemsex and the development of psychosis is particularly relevant in light of the results of a systematic review and meta-analysis, which showed that psychotic patients are at significantly increased risk for suicidal ideation, as well as suicide (attempts) [21]. Especially in view of the previously evidenced increased prevalence of mental health disorders among LGB people, the frequently demonstrated connection between sexualized substance use and mental burden, and the significantly increased risk of suicidal ideation and suicides in patients with psychoses [21], an association between the practice of chemsex and suicidality appears likely. However, to the best of our knowledge, no review thus far has investigated the possible relationship between chemsex and suicidality. Moreover, while there appear to be numerous studies on the association between chemsex and physical health, such as STIs, there seems to be very little research focusing on mental health in general.

Overall, whilst taking into consideration the potential risk factors, mental health among LBTQ individuals performing chemsex has been insufficiently addressed in research, especially research about the issue of suicidality. Therefore, the aim of the present review is to address this knowledge gap by investigating whether the psychological burden of MSM who combine chemsex drugs with sex includes suicidal behavior in particular. Our narrative review was conducted focusing on the following question:

Is the practice of chemsex associated with an increased risk of suicidal ideation, suicide attempt, and/or suicide death?

## 2. Methods

In contrast to systematic reviews, narrative reviews do not follow an explicit methodology with regard to the selection, extraction, and synthesis of data [22]. However, for the sake of comprehensibility of the literature search and the inclusion of studies and in order to minimize selection bias, the methodological steps of the present narrative review are presented below. As suggested by Ferrari and Gasparyan et al. [22,23], we first developed a search term that included all the keywords relevant to our research question and defined inclusion criteria for literature selection. Subsequently, all the articles identified through the search were evaluated based on their suitability for our review by considering the fit of the respective study’s objectives, the methods used, the quality of the results, and the limitations. As a final step, according to Ferrari, the included studies should be synthesized to detect and highlight potential inconsistencies among the results. 

For the purpose of this narrative review, we searched Pubmed for the terms “chemsex”, “sexualized drug use”, “slamsex”, “suicide”, and “mental health”. The literature search was conducted in September 2022. In total, we retrieved 52 articles published between 2016 and 2022. Due to the novelty of chemsex as a topic in scientific research, no studies conducted prior to 2016 were found in our search. The reference lists of relevant papers were screened to find additional articles. A paper that was known to the authors but not found by the search was included. The titles and abstracts were screened for relevance by two independent researchers, and full texts of eligible articles were retrieved. Studies were included when they (a) were original studies, (b) were published in English, (c) were focused on a study population that included chemsex users (following the expanded chemsex definition), and (d) made at least one statement about suicidal behavior among the study participants.

## 3. Results

From the initial 53 articles captured through the search, 12 eligible articles were finally identified and included in the synthesis. A summary of the characteristics of each study is provided in Table 1. Except for one study from Singapore [24], all data were published in Western cultures (e.g., Europe, USA, and Canada). The studies retrieved included three case reports [25,26,27] and nine cross-sectional studies [24,28,29,30,31,32,33,34,35], the results of which are presented below, following the type of the study.

### 3.1. Case Reports

We identified three case reports about men who were hospitalized in the course of a suicidal crisis [26,27] and/or a severe psychosis [25,27] after practicing chemsex sessions almost every weekend. For all users, the administration of chemsex-related drugs (mephedrone and methamphetamine) was primarily intravenous. Regarding suicidal behavior, two of the patients showed acute suicidal ideation and behavior at the time of admission into a psychiatric unit [26,27]. The third patient had a reported history of suicide attempts by drug intoxication [25]. The authors described an improvement and, finally, the disappearance of depressive symptoms [27], psychotic symptoms [25], and chemsex behavior [26] after adjusted medical treatment [25,27] or transcranial direct current stimulation, respectively [26]. Garcia-Fuentes et al. reported a decrease in suicidal ideation in their participant explicitly [27]. The other articles did not provide information about further clinical effects of suicidal ideation in particular.

### 3.2. Cross-Sectional Studies

Within the nine cross-sectional studies, participants were recruited from both drug service/sexual health/HIV clinic settings [28,29,31,32,35] and the general community via online questionnaires [24,30,33,34]. There was much heterogeneity between the study populations: In one of the analyzed studies, both MSM and men who have sex with women (MSW) were included [35]. Another study included only slamming chemsex users [29]. Two other studies included only HIV-negative MSM [24,31] while one included only HIV-positive MSM [32]. The reported chemsex prevalence in the study populations ranged between 4% [24] and 100% [29,31], considering all the articles included in the review. Regarding the studied period of time, chemsex practice was analyzed by two studies over a lifetime [24,33]. Four studies reported the prevalence of engaging in chemsex in the last 12 months [30,32,34,35] and one in the previous 6 months [33]. Three studies did not mention any information about the observation period [28,29,31]. All studies included at least two of the three chemsex-related drugs in the narrower sense: Methamphetamine, GHB/GBL, and mephedrone. Some of the analyzed studies described further substances used by chemsex participants, including ketamine, cocaine, MDMA, amphetamine, poppers, and erectile dysfunction drugs. A more detailed overview of investigated substances can be found in Table 2. The most common method of administration—when indicated—was non-intravenous. In five studies, drugs were also injected [28,29,31,32,35]. Explicit data on the frequency of engaging in chemsex and the dosages of the consumed substances could not be found.

Regarding data assessment techniques, self-administered online questionnaires were applied in five studies [24,30,32,33,34] and a self-administered paper-and-pencil questionnaire was applied in one study [35]. Two studies used oral interviews [28,31], and one used clinical observations recorded in notifications [29]. In most studies analyzed, suicidal symptoms were self-reported via questions about previous suicidal thoughts or attempts while two studies assessed suicidal tendencies using standardized instruments including the Hopkins Symptom Check List (HSCL-10) [35] and the first question of the Suicide Behaviors Questionnaire-Revised [34]. With regard to the period of time studied, suicidal ideation and behaviors were analyzed by three studies over a lifetime [24,30,34], one in the previous 12 months [35], and one in the previous two weeks [33]. One study assessed the prevalence of previous suicidal ideation and attempts during or right after chemsex in the context of withdrawal and intoxication-related symptoms [32]. Three of the analyzed articles did not provide detailed information about the time period of suicidal symptoms studied [28,29,31]. All studies assessed the prevalence of suicidal behaviors in parts of their study population (for details see Table 2). Eight of the studies analyzed assessed further aspects of perceived mental health. The most frequent outcomes were depressive symptoms [24,30,32,34], anxiety symptoms [30,32,34], combined measurements of depression and anxiety symptoms [33,35], and self-reported mental health diagnoses [29,31].

Except for one study, which did not find significant differences in depression and anxiety outcomes between groups [30], all studies reported a higher mental health burden for those engaging in chemsex compared to the respective control group [24,34,35]: Berg et al. noted that chemsex users had two times higher odds of reduced mental health [35]. Moreover, a positive association between chemsex practice and depressive symptoms was revealed by two studies [24,34] while a positive association between chemsex practice and anxiety symptoms was found in one study [34]. According to Dolengevich-Segal et al., those chemsex users who inject drugs were more likely to experience depression and anxiety symptoms than participants who engaged in non-injecting chemsex [32]. Regarding STIs, the most frequent outcome was patients’ HIV status, which was assessed in every eligible article, while the association between HIV status and chemsex was assessed in three out of nine [28,30,34]. In these studies, chemsex participants showed significantly higher HIV prevalence compared to the respective control groups. Furthermore, in two [30,34] out of the three studies, the chemsex group showed reduced mental health compared to the respective control groups. Explicit data on the comorbidity of depression and suicidality among chemsex users—especially in the sense of a comparison of depressed and non-depressed chemsex users—could not be found.

A total of six studies reported the prevalence of suicidal ideation in chemsex users [24,29,30,31,32,34] and four of these studies provided data on the prevalence of suicide attempts [30,31,32,34]. Estimates of the prevalence of reported suicidal ideation and attempts ranged from 11% [29] to 83% [24] and from 7.1% [30] to 27% [31], respectively. Three studies did not provide data on the prevalence of suicidal behavior among chemsex users in particular but only on the entire sample [28,33,35].

Among those studies that compared suicidality among chemsex users and MSM who did not engage in chemsex, three of the eligible studies found no significant relationship between chemsex practice and suicidality outcomes [30,34,35]. Stevens et al. [28] established a negative association between practicing chemsex and suicidal ideation. Those MSM seeking assistance for chemsex-related drug use had a decreased risk of suicidal ideation compared to those who presented for problematic use of other drugs, largely alcohol and cocaine [28]. The authors highlighted that their selection of the control group, including MSM with problematic cocaine and alcohol use, may have led to a higher control prevalence of suicidal ideation than in the wider MSM population; this may, at least in part, explain this uncommon finding [28]. Moreover, those selecting mephedrone as a drug of choice were less likely to experience past suicidal ideation [28]. In contrast, Tan et al. [24] indicated that there was a positive relationship between chemsex and suicidality outcomes. Compared to MSM who consumed alcohol or primarily poppers, chemsex users were more likely to report a history of suicidal ideation [24]. Moreover, Scholz-Hehn et al. [30] used a latent class analysis and identified four subgroups based on the consumed substances. Those MSM who reported a wide range of consumed substances, including but not limited to chemsex-related drugs, had a significantly higher lifetime prevalence of suicide attempts compared to MSM from other clusters [30]. Finally, regarding the form of consumption, Dolengevich-Segal et al. [32] found that MSM who engaged in slamsex experienced more severe psychopathological symptoms, including suicidal behaviors, compared to MSM who did not inject drugs. No further characteristics of those chemsex users who might experience adverse mental health symptoms were mentioned in the articles.

Table 2 provides a summary of the study population, sample size, age, drugs of concern, key findings, and, if indicated, a statement on suicidal behavior and chemsex of each study.

## 4. Discussion

Growing evidence shows that MSM who engage in chemsex may be a small but especially vulnerable group of men regarding both physical harm and adverse mental health outcomes. To further evaluate this, we reviewed the recent literature to explore whether suicidality among chemsex users has been sufficiently addressed in research. In total, we identified twelve studies of interest, including three case reports and nine cross-sectional studies that made at least one statement on the presence of suicidal symptoms in chemsex users.

In the analyzed case reports, three slamming chemsex users were described. They showed depressive symptoms [26,27], drug dependence, psychotic symptoms [25,27], and, finally, suicidal symptoms in the context of escalating slamsex sessions. Further, the authors noted an improvement in mental health conditions and/or the disappearance of chemsex behaviors after a few weeks of admission to a psychiatric ward, adjusted medical treatment, and/or daily sessions of prefrontal cortex stimulation, respectively. So far, there is very limited research on slamming chemsex and suicidal symptoms. In their review, Íncera-Fernández et al. [18] investigated the association between intravenous drug use and mental health. They selected three relevant studies (one is also included in the present review) that suggested that slamsex participants develop mental health symptoms such as depression or anxiety more frequently compared to those MSM who do not inject drugs. In summary, these case reports support the assumption that suicidality could be a symptom of concern in chemsex users. 

Within the quantitative studies analyzed, the prevalence of suicidal symptoms in chemsex users, when indicated, ranged widely. This can partly be explained by the differences in study designs and outcomes. The findings of the studies that investigated the association between chemsex practice and suicidal symptoms were contradictory. For the majority of the studies, the chemsex group did not differ significantly from the respective control group regarding suicidal ideation and attempts [30,34,35]. Interestingly, Stevens et al. found a negative association between chemsex practice and suicidal ideation, indicating chemsex users experienced less frequent suicidal ideation than the control group [28]. In contrast, Tan et al. revealed a positive association and noted that suicidal ideation was more frequently observed in those who practiced chemsex [24]. Several factors might underlie these inconsistent findings. With regard to our main subjective interest, namely addressing suicidal symptoms among MSM who combine sex with drugs, the studies used a variety of types of study populations and sampling techniques. Some studies were conducted in the general population [24,30,33,34] and some in HIV/sexual health clinics or alcohol and drug services [28,29,31,32,35]. Regarding the sample populations, one study included both MSM and MSW [35], while other studies were restricted to HIV-positive [32] or HIV-negative men [24,31]; only one studied intravenous drug users [32]. This creates much heterogeneity in the study populations, which makes comparisons between studies difficult. As mentioned before, Stevens et al., for example, found that chemsex users were less likely to report past suicidal ideation as compared to the control group [28]. Here it should be emphasized that the control group comprised MSM who were themselves seeking assistance for problematic drug use, including alcohol and cocaine in contrast to the other studies included in this review [28]. Therefore, baseline measurement differed clearly, which might be an explanation for this particular finding. Within the articles that did not find a relationship between chemsex practice and suicidal ideation, Scholz-Hehn et al., for example, recruited MSM for an anonymous online survey and conducted a cluster analysis based on the consumed substances [30]. It was not the chemsex group (comprised of MSM who used primarily chemsex-related drugs), but rather the group of polyvalent drug users (comprised of MSM who reported use of a wide range of substances, including chemsex-related drugs) that reported a significantly higher prevalence of lifetime suicide attempts [30]. These results are in line with the findings of Maxwell et al., who noted that, although evidence for the impact of chemsex behaviors on psychological well-being may be weak, polydrug users suffer more frequently from both physical and mental illnesses [5]. Therefore, another explanation for the inconsistent findings of the studies is likely due to the absence of a consensus on the term “chemsex” and the substances investigated in this context. Study results would, therefore, generally benefit from a sharper definition of polyvalent substance users without chemsex behavior, chemsex users with a broader substance use pattern, and chemsex users following the classical definition.

Finally, it should be mentioned that the country setting might also play an important role in the mental health of chemsex participants. While all other studies selected were published in Western cultures, Tan et al. conducted a web-based survey among self-identified HIV-negative young MSM in Singapore [24]. There, the possession and consumption of drugs are criminalized and accompanied by severe penalties. Although the survey was conducted anonymously, participants might not be entirely honest with their answers about substance use [24]. As expected, the chemsex group was relatively small and the prevalence of mental health symptoms including suicidal ideation was high. Additionally, in Singapore, MSM are confronted with stigma emanating from society, and sexual relations between men are criminalized by law [24]. This may lead to further mental burden and is aggravated by the absence (or use) of support and information services, showing that studies from other cultural backgrounds have to be understood in a specific societal context and results cannot simply be generalized to other populations.

Overall, while not every kind of chemsex seems to be problematic in nature, our findings indicate that suicidal ideation and attempts might play a role in some persons with sexualized substance use. We found a prevalence of reported suicidal ideation ranged from 11% [29] to 83% [24] and from 7.1% [30] to 27% [31] for suicidal attempts among chemsex users. A recent meta-analysis [36] provided data on the lifetime prevalence of suicidal ideation among men who have sex with men. Similar to our findings, Luo et al. observed a high degree of heterogeneity among the eligible studies. Estimates of the reported lifetime prevalence of suicidal ideation among MSM ranged from 13.2% to 55.8%, and the pooled lifetime prevalence was 35% [36]. This pooled lifetime prevalence was markedly higher than the prevalence estimates of lifetime suicidal ideation among the general population (3.9–20.5%) [37,38]. Various factors, such as discrimination, stigmatization, prejudice, and isolation on the basis of the sexual orientation of those affected, might explain this higher vulnerability [8,39]. Furthermore, the authors noted an association between suicidality outcomes and HIV status [36]. The lifetime prevalence of suicidal ideation was much higher in HIV-positive MSM compared to HIV-negative MSM [36]. As mentioned above, previous research suggested a complex interplay between substance use, sexually high-risk behavior, and mental health measures. Some evidence indicates that high risk chemsex behaviors, such as condomless anal intercourse, put participants at increased risk of acquiring HIV and other STIs [5]. In our review, three studies that compared the HIV status of chemsex users and non-chemsex users could be identified [28,30,34]. Men in the chemsex group showed significantly higher HIV prevalence rates. Moreover, in two out of three studies, chemsex and polyvalent drug users had impaired mental health when compared to the respective control groups [30,34]. The higher rates of HIV-positive chemsex users in these studies allow us to conclude that the mental health of chemsex participants may be negatively impacted by HIV and other STIs. Based on the presented scientific evidence, an additive effect on the mental health of chemsex participants, consisting of the experienced adversities due to one’s sexual orientation and the negative impact caused by HIV and other STI infections, seems likely. Further, other risk factors for suicidality not limited to the group of those practicing chemsex, such as depression, hopelessness, or the presence of an abuse history [40], may be partly accountable for the heterogeneous results reported in this review. However, this warrants further research. Other factors that might impact chemsex users’ mental health include consumption behavior. Unfortunately, no detailed information about the dosages of consumed substances, the frequency of engaging in chemsex, or the samples’ rate of substance dependency could be found in the eligible articles.

### 4.1. Strengths and Limitations

A strength of the current review is that it addresses a research gap, namely suicidal symptoms among chemsex users. To our knowledge, it is the first study of its kind. Nevertheless, our review has several limitations. Results must be interpreted cautiously because of the inhomogeneous nature of the studies regarding chemsex definition, study populations, and study methodology. Further limitations include that we focused on Pubmed/MEDLINE and did not search other databases. Moreover, we were not able to provide a quantitative analysis due to the heterogeneous and incomparable nature of the included studies. In that regard, our narrative review does not fully adhere to the recommendation of PRISMA (Preferred Reporting Items for Systematic Reviews and Meta-Analyses). Therefore, these aspects warrant further investigations with a clear chemsex definition over a certain period, a detailed assessment of the consumed substance dosages and the frequency of use, a larger study sample and a sharp definition of chemsex users following the classical definition, chemsex users with a broader substance use pattern, and polyvalent substance users without chemsex behavior. Furthermore, longitudinal studies are needed to clarify the issue of causality, which cannot be drawn from the studies included in this review. Overall, there is a clear need for further research on the mental health of lesbian, gay, bisexual, transgender, queer, intersexual, and other (LGBTQI+) people, and a clear need for further professional support and care for affected individuals.

### 4.2. Conclusions

The association between chemsex and suicidal symptoms is poorly investigated. According to the data obtained, the prevalence of suicidal ideation and attempts are markedly higher among the MSM population as compared to the general population and might be even further elevated in MSM who engage in chemsex. However, we cannot draw a direct association between sexualized drug use and suicidal behavior as the evidence is limited and partly contradictory. Therefore, further research is required to clarify the issue of causality and to determine the characteristics of chemsex users who experience adverse mental health effects and establish effective support and care for affected individuals.

## Figures and Tables

**Table 1:** Descriptive characteristics of included studies.

Author and Year	Title. *Journal* (DOI)	Country	Study Design	Objective of the Study
Berg et al., 2020 [35]	Links between chemsex and reduced mental health among Norwegian MSM and other men: Results from a cross-sectional clinic survey. *BMC Public Health* (10.1186/s12889-020-09916-7)	Norway	Cross-sectional	To investigate the link between chemsex and mental health among MSM.
Bohn et al., 2020 [34]	Chemsex and Mental Health of Men Who Have Sex With Men in Germany. *Frontiers in Psychiatry* (10.3389/fpsyt.2020.542301)	Germany	Cross-sectional	To describe aspects of mental health of MSM who engage in chemsex and to describe potentially adverse consequences of chemsex behavior.
Brogan et al., 2019 [33]	Canadian results from the European Men-who-have-sex-with-men Internet survey (EMIS-2017). *Canada Communicable Disease Report* (10.14745/ccdr.v45i11a01)	Canada	Cross-sectional	To assess needs related to sexually transmitted infections of gays, bisexuals, and other MSM.
Dolengevich-Segal et al., 2019 [32]	Drug-related and psychopathological symptoms in HIV-positive men who have sex with men who inject drugs during sex (slamsex): Data from the U-SEX GESIDA 9416 Study. *PLOS ONE* (10.1371/journal.pone.0220272)	Spain	Cross-sectional	To describe the physical and psychopathological symptoms of sexualized intravenous drug use (slamsex).
Dolengevich-Segal et al., 2016 [25]	Severe Psychosis, Drug Dependence, and Hepatitis C Related to Slamming Mephedrone. *Case Reports in Psychiatry* (10.1155/2016/8379562)	Spain	Case report	To describe the case of a patient who developed psychotic symptoms after months of slamming mephedrone.
Garcia-Fuentes et al., 2022 [27]	Attempted suicide with intravenous methamphetamine and chemsex. *Revista Colombiana de Psiquiatría (English ed.)* (10.1016/j.rcpeng.2020.09.002)	Spain	Case Report	To report a case that encompasses polysubstance use disorder, chemsex, psychotic symptoms, undiagnosed ADHD, and depressive disorder with a suicide attempt.
Malandain et al., 2020 [26]	First case report of tDCS efficacy in severe chemsex addiction. *Dialogues in Clinical Neuroscience* (10.31887/DCNS.2020.22.3/lmalandain)	France	Case report	To report the case of a patient with severe chemsex addiction who was treated with tDCS.
Maxwell et al., 2020 [31]	Pre-exposure prophylaxis use among men who have sex with men who have experienced problematic chemsex. *International Journal of STD & AIDS* (10.1177/0956462420906927)	United Kingdom	Cross-sectional	To examine the biopsychosocial characteristics associated with PrEP use among HIV-negative MSM who have experienced problematic chemsex.
Scholz-Hehn et al., 2022 [30]	Substance Use and Chemsex in MSM—A Latent Class Analysis. *Journal of Drug Issues* (10.1177/00220426211040564)	Germany	Cross-sectional	To identify subgroups based on the consumed substances and to assess their psychosocial and health-related characteristics, including sexual risk behaviors.
Schreck et al., 2020 [29]	Cathinone Use Disorder in the Context of Slam Practice: New Pharmacological and Clinical Challenges. *Frontiers in Psychiatry* (10.3389/fpsyt.2020.00705)	France	Cross-sectional	To characterize cathinone use disorder through analyses of slam cases and to expand the knowledge of slam practice based on data on drug use, risk taking, and harmful consequences.
Stevens et al., 2020 [28]	Chemsex-related drug use and its association with health outcomes in men who have sex with men: A cross-sectional analysis of Antidote clinic service data. *Sexually Transmitted Infections* (10.1136/sextrans-2019-054040)	UK	Cross-sectional	To assess associations between CDU and a range of health outcomes and to determine whether MSM seeking assistance for CDU are a higher risk population than MSM presenting for any other drugs.
Tan et al., 2021 [24]	Delineating patterns of sexualized substance use and its association with sexual and mental health outcomes among young gay, bisexual and other men who have sex with men in Singapore: A latent class analysis. *BMC Public Health* (10.1186/s12889-021-11056-5)	Singapore	Cross-sectional	To classify patterns of sexualized substance use among young MSM, and to investigate its association with sexual and mental health outcomes.

Synopsis of the included studies with details about the authors, the title, country of origin, study design, and objective. Abbreviations: Chemsex-related drug use (CDU); Human immunodeficiency virus (HIV); Men who have sex with men (MSM); Pre-exposure prophylaxis (PrEP); Transcranial direct current stimulation (tDCS).

**Table 2:** Summary of findings.

Author and Year	Study Population	Sample Size (N)	Chemsex Users (%)	Age	Drugs Included in Chemsex Definition of the Study	Main Results	Statement on Suicidal Behavior and Chemsex
Berg et al., 2020 [35]	Male attendees of an STI clinic (51% MSM)	1013	14	Mean = 33	Cocaine, GHB/GBL, methamphetamine, ketamine, mephedrone	21% * of the men had reduced mental health. Chemsex users (slightly more MSM), had two times greater odds of reduced mental health.	Suicidal ideation ^b^: 3.3% * Suicide attempts ^b^: 0.5% * No difference between those who engaged in chemsex and those who did not
Bohn et al., 2020 [34]	MSM recruited via LGBT-related platforms, websites, social media channels and HIV/sexual health clinics	1050	27	Mean = 40.2	Methamphetamine, mephedrone, GHB/GBL, ketamine	The chemsex group showed significantly higher mean scores for depression, anxiety, and somatization, but the effect sizes were low. Mean scores were below the cut-off for clinically relevant symptoms.	Suicidal ideation ^a^: 12.7% ** Suicide attempts ^a^: 9.7% ** No difference between the chemsex and non-chemsex groups
Brogan et al., 2019 [33]	MSM recruited via gay-related “dating” platforms, websites, social media, and community-based organizations	5165	21.5	Median = 36	Ecstasy/MDMA, cocaine, amphetamine, methamphetamine, mephedrone, ketamine	Participants experienced high levels of intimidation, verbal abuse and physical violence related to their sexual orientation. Mental health problems, substance use, high-risk sexual practices were high. Only few men actually used PrEP. The comprehensive STBBI testing was low.	Suicidal ideation ^c^: 26.1% * No data on suicidal ideation in chemsex users in particular
Dolengevich-Segal et al., 2019 [32]	HIV-positive MSM recruited from HIV clinics	742	29.1	Median = 38	Mephedrone, MDMA, methamphetamine, GHB/GBL, ketamine, cocaine	Slamsex is associated with current psychiatric disorders, high-risk sexual behaviors, polydrug use, diagnosis of STIs, and severe drug-related and psychiatric symptoms.	Suicidal ideation: 15.3% ** Suicide attempts: 13.8% ** Slamsex user experienced more suicidal behaviors.
Dolengevich-Segal et al., 2016 [25]	Patient of a psychiatric ward	1	100	25	Mephedrone	The psychotic symptoms gradually began to improve and disappeared after four weeks of treatment.	Patient was treated in the emergency room after attempting suicide by drug intoxication.
Garcia-Fuentes et al., 2022 [27]	Patient of a psychiatric ward	1	100	44	Methamphetamine, mephedrone	After his psychiatric drug treatment was adjusted, the patient showed gradual affective improvement.	Patient presenting with 2nd time suicidal high-risk attempt. After the treatment, suicidal ideation ceased.
Malandain et al., 2020 [26]	Patient of a psychiatric ward	1	100	43	Synthetic cathinones (3-MMC, mephedrone)	Chemsex behavior disappeared after 5 days of daily sessions of right prefrontal cortex stimulation and did not return after 8 months of follow-up.	Patient was hospitalized in the context of a suicidal crisis.
Maxwell et al., 2020 [31]	HIV-negative MSM recruited from an alcohol and drug service	165	100	Median = 36	Methamphetamine, GHB/GBL, mephedrone, cocaine, ketamine	34% had ever used PrEP. These were men who had engaged in higher risk sexual behaviors than men who had never used PrEP.	Suicidal ideation: 49% ** Suicide attempts: 27% ** No difference between those who ever used PrEP and those who never used PrEP.
Scholz-Hehn et al., 2022 [30]	MSM recruited via websites and social media channels directed at gay community members	597	7.2	Mean = 38.2	Methamphetamine, GHB/GBL, ketamine, mephedrone	LCA revealed four different clusters: Use of mainly (1) amyl nitrite and cannabis, (2) MDMA, cocaine and amphetamine, (3) chemsex-related drugs, (4) polyvalent drugs.	Suicidal ideation: 23.7% *, 31% ** Suicide attempts^a^: 9.5% *, 7.1% ** Clusters did not differ in the percentage of suicide ideation. Prevalence of suicide attempts was higher in the polyvalent cluster.
Schreck et al., 2020 [29]	39 Notifications of MSM by health professionals collected at an addictovigilance center	34	100	Median = 38	Synthetic cathinones, GHB/GBL, poppers, cocaine	39 slam notifications were collected. The severity of cathinone use disorder was mild, moderate, and severe for 18%, 12%, and 58% of the patients, respectively.	Suicidal ideation: 11% **
Stevens et al., 2020 [28]	MSM recruited from an LGBT drug and alcohol service (Antidote)	2137	88	Median = 35.2	Mephedrone, GHB/GBL, methamphetamine	MSM seeking assistance for chemsex-related drug use are a high-risk population. Alcohol is an underappreciated drug of concern.	Suicidal ideation: 15% * Presenting for CDU is associated with a decreased risk of suicidal ideation. Those selecting mephedrone are less likely to have suicidal ideation.
Tan et al., 2021 [24]	Young HIV-negative MSM recruited via online (e.g., social media) and offline (e.g., community-based organizations) channels	570	4	Mean = 21.9	Methamphetamine, GHB/GBL, erectile dysfunction drugs	LCA revealed three clusters: Use of (1) only alcohol, (2) primarily amyl nitrite, and (3) mostly chemsex-related drugs. Those in the chemsex group were more likely to report poorer sexual and mental health outcomes.	Suicidal ideation ^a^: 54% *, 83% ** Participants who were in the chemsex class were more likely to report a history of suicide ideation.

Synopsis of the general findings of the studies included in this review and specific statement concerning suicidality concluded by the authors. Note: * Prevalence for the entire sample; ** Prevalence for chemsex users; ^a^ over lifetime; ^b^ in the previous 12 months; ^c^ in the previous two weeks; Abbreviations: Chemsex-related drug use (CDU); Gamma hydroxybutyrate/Gamma hydroxybutyric (GHB/GBL); Human immunodeficiency virus (HIV); Latent class analysis (LCA); lesbian, gay, bisexual and transsexual (LGBT); Methylenedioxymethamphetamine (MDMA); Men who have sex with men (MSM); Pre-exposure prophylaxis (PrEP); Sexually transmitted and other bloodborne infection (STBBI); Sexually transmitted infections (STI).

## Data Availability

Not applicable.

## Abbreviations

Chemsex-related drug use (CDU), gamma-butyrolactone (GBL), gammahydroxybutyrate (GHB), Hopkins Symptom Check List (HSCL-10), Human immunodeficiency virus (HIV), latent class analysis (LCA), lesbian, gay and bisexual (LGB), lesbian, gay, bisexual, transsexual, queer, intersexual and other (LGBTQI+), Methylenedioxymethamphetamine (MDMA), men who have sex with other men (MSM), men who have sex with women (MSW), Pre-exposure prophylaxis (PrEP), sexually transmitted and other bloodborne infection (STBBI), Sexually Transmitted Infections (STI), Transcranial direct current stimulation (tDCS).
